# Detection of Nickel Atoms Released from Electrodes in Spark Discharges Using Laser-Induced Fluorescence

**DOI:** 10.1177/00037028241285150

**Published:** 2024-09-28

**Authors:** Kailun Zhang, Ruike Bi, Johan Tidholm, Jakob Ängeby, Mattias Richter, Andreas Ehn

**Affiliations:** 1 Division of Combustion Physics, Department of Physics, 5193 Lund University, Lund, Sweden; 2 Swedish Electro Magnets (SEM) AB, Åmål, Sweden

**Keywords:** Spark plug, electrode wear, nickel atom, laser-induced fluorescence, LIF‌, spectroscopy

## Abstract

The reduction of greenhouse gas emissions and the effort of carbon neutrality require the improvement of spark-ignition engines in terms of efficiency and capability to operate on renewable fuels. The electrode wear of spark plugs, used for ignition of novel fuels and lean mixtures, emerges as a significant challenge in this transition. Understanding the physical mechanism and influence of spark operation parameters of the wear process is thus important. Compared to the conventional methodology of performing long-term wear tests, laser-based optical diagnostics methods are capable of assessing electrode wear during one single or a few spark discharges. In this work, the necessary initial steps required for performing optical investigations using laser-induced fluorescence (LIF) are presented. Several excitation pathways of nickel atoms were investigated, and 336.96 nm was identified as the optimal one. This excitation approach yielded emissions between 338.75 and 353.58 nm, effectively avoiding the major interference from N_2_ plasma emission in spark discharges. Additionally, a linear relationship in fluorescence signal intensity with excitation energy up to 400 µJ was observed. These findings indicate the potential of LIF for in situ diagnostics of electrode wear, contributing to engine development in both efficiency and compatibility with sustainable fuels.

## Graphical abstract

**Graphical abstract:**
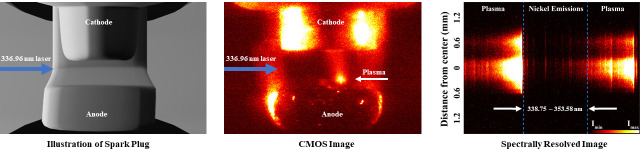
This is a visual representation of the abstract.

## Introduction

To achieve the European Union's mid-term goal of greenhouse gas emission reduction, i.e., a 40% reduction by 2030 compared with the level of 1990^1^ and even climate neutrality in 2050,^2^ improvement of spark-ignition engines for more efficient combustion and adaptation to renewable fuels becomes more important or even necessary.^3,4^ However, in the field of transportation, electrode wear of spark plugs is a growing problem, especially in engines designed for lean operation on renewable fuels with high efficiency and high in-cylinder pressures. The high-performance ignition systems designed for such applications nowadays lead to an increase in spark plug wear.^5^ When the operation lifetime of the spark plugs falls shorter than the vehicle's normal service intervals, undesired downtime or even damage to the engine can occur.^6^

The conventional methodology for investigating spark plug wear is to run long-term tests in engines and examine the loss of electrode material visually.^5–11^ Such data are valuable, but, needless to say, it is both time and resource-consuming since the gradual degradation of ignition performance is a long-term process, while the actual ignition process is over within just about one to several milliseconds (ms). To obtain the required knowledge of the relationship between electrode wear and operating parameters of spark discharges, the wear process must hence be examined with extremely high temporal resolution.

Laser-based spectroscopic diagnostic techniques have been widely applied to investigate complicated and difficult-to-access processes with high temporal and spatial resolutions, such as combustion and plasma.^12–15^ Among them, laser-induced fluorescence (LIF), known for its high selectivity and sensitivity, is capable of detecting trace amounts of species even under conditions of strong background interference. Different metal elements have successfully been monitored and measured in lots of studies using LIF inside an inductively coupled plasma mass spectrometer.^16–24^ While, for nickel (Ni), the common base metal material for spark plug electrodes,^25^ only several studies have been performed using LIF. Lins and Hartmann^26^ measured the number density of Ni in the pseudo spark switch. Marunkov et al.^27^ detected trace amounts of nickel in a graphite furnace. As a technique for elements analysis, Epstein et al.^28^ applied LIF to determine nickel in different matrices, and Li et al.^29^ and Li^30^ used LIF to enhance the excitation efficiency of nickel atoms in laser-induced breakdown spectroscopy. Some studies investigated nickel as the metal catalyst with LIF in reactors during carbon nanotube formation.^31–34^ Among all the aforementioned studies, only Marunkov et al.^27^ discussed the choice of excitation wavelengths of nickel theoretically. Thus, a systematic experimental investigation needs to be conducted on both the excitation and emission sides of laser-induced nickel fluorescence for studying electrode wear.

**Figure 1.**
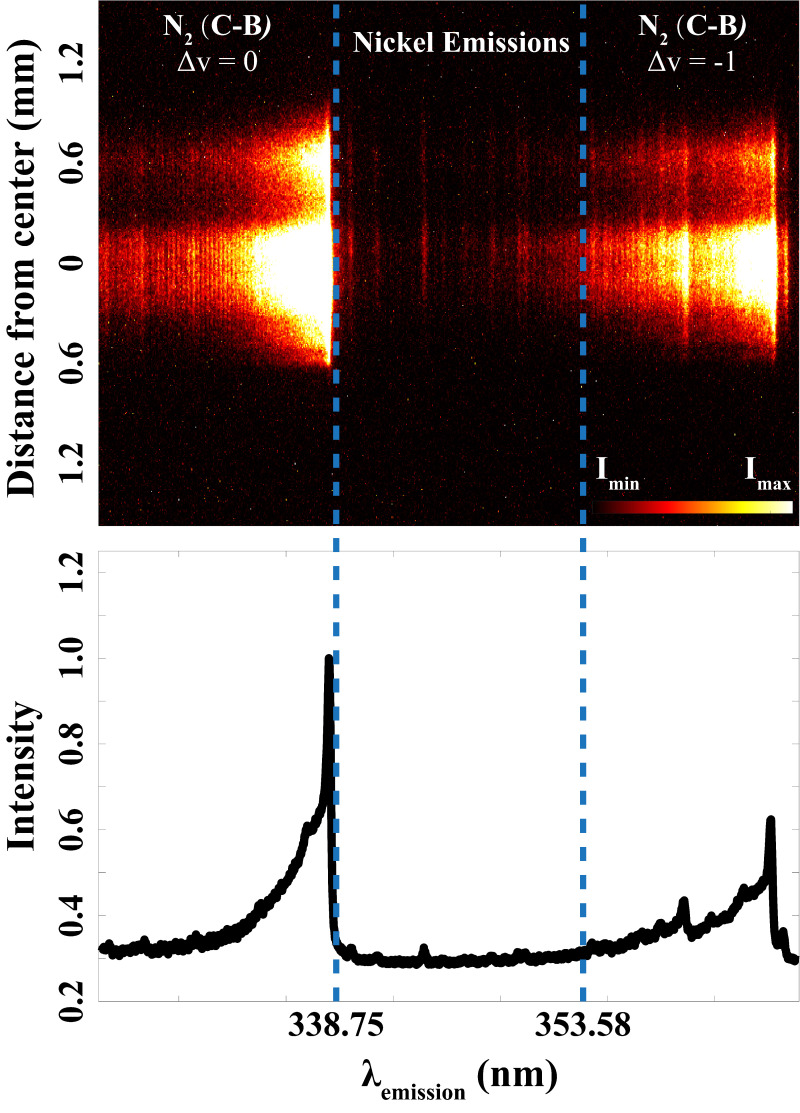
Nitrogen (N_2_) plasma emissions (outside two blue dashed lines) and plasma-induced emissions of nickel atoms (between two blue dash lines) in spark discharges.

A previous spectral investigation of emissions from spark discharges by Kim et al.^35^ shows that several metal atomic lines from nickel, Ni(I), exist in the gap between the two strong nitrogen (N_2_) emission bands, whose bandheads are located at 337.1 nm (C^3^Π_u_ → B^3^Π_g_, Δv = 0) and 357.6 nm (C^3^Π_u_ → B^3^Π_g_, Δv = –1).^36^ Different from the wide spectral range they studied, Figure 1, recorded in the present study, shows the spectrally resolved emission in spark discharges specifically focused on this narrow region. Seen outside the two blue dashed lines, due to the luminous plasma and thus the intense background emission from the discharge, it is very challenging to do qualitative and quantitative detections of the metal atoms that stem from the electrodes. With the help of laser-based optical methods, using pulsed excitation and detection, such intense background emissions can be minimized.

This work outlines the necessary initial steps for utilizing laser-based spectroscopic diagnostic techniques for studying electrode wear. A strategy is presented and demonstrated for detection of evaporated metal atoms when sparks transfer energy to the electrodes. The LIF from nickel atoms in atmospheric pressure conditions was captured. Various excitations and subsequent emissions are investigated and assessed to gain knowledge about suitable transitions for laser-based optical measurements. Moreover, a dependence of fluorescence intensity on excitation laser energy was also studied.

## Experimental

### Materials and Methods

**Figure 2.**
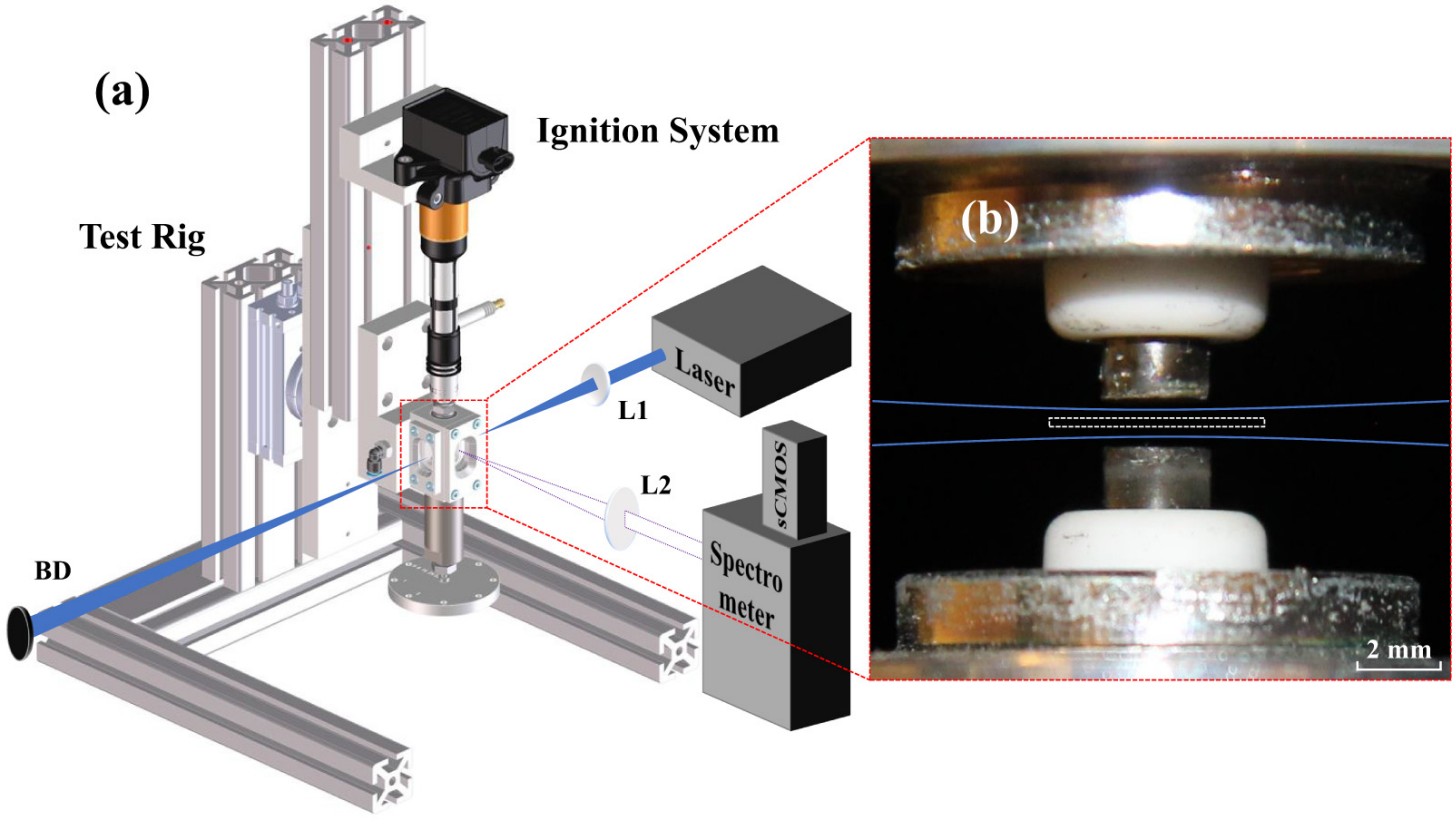
(a) Illustration of the experimental setup. (b) An image of the sparkplugs with relative positions of the excitation laser (blue curves) and the entrance slit of the spectrometer (white dashed box).

A schematic illustration of the experimental setup is shown in Figure 2a. It consists of a tunable picosecond laser system (Ekspla UltraFlux FT405), an ignition system (Swedish Electro Magnets SEM Ignition System), a spectrometer (Princeton Instrument Acton SpectraPro 2300i) attached to a complementary metal oxide semiconductor (CMOS) camera (Oxford Instruments Andor iStar sCMOS), and light-guiding optics. A digital delay generator (Stanford Research Systems DG535) was used to synchronize all the equipment.

### Optics

The laser system was operating at a repetition rate of 5 Hz, generating tunable laser pulses with a pulse duration of ∼90 ps with a pulse energy of 400 μJ. The line width, full width half-maximum (FWHM), of the laser is about 9 cm^–^^1^, which is ∼0.1 nm at 337.0 nm. The laser was focused into the probe volume with a fused-silica lens (L1) which has a 500 mm focal length and was carefully terminated by a beam dump to minimize interfering laser reflections.

The ultraviolet signal was collected by a fused silica lens (L2, B. Halle, *f *= 100 mm) with a large aperture (*f*/2) and focused onto the entrance slit of the spectrometer. The grating of the spectrometer had 1200 lines per millimeter and the width of the entrance slit was tuned to 0.1 mm to obtain a sufficiently high spectral resolution and allow enough signal intensity for collection. The CMOS camera is equipped with a Gen-II image intensifier with a relatively high quantum efficiency (>20%) in the 250–350 nm region, where the LIF signal was expected. Also, to eliminate the possible interference from the two emission peaks originating from nitrogen, the grating of the spectrometer was centered at 345.5 nm to cover the range from 338.75 to 353.58 nm after calibration.

The gate of the camera was fixed at 300 μs after the breakdown of the discharges to maximize the signal from nickel atoms.^35^ Meanwhile, to diminish the background of plasma emission, the exposure time was set to the lowest limit, which is about 2.1 ns. The fluorescence signal of 100 sparks is accumulated by software for a decent signal-to-noise ratio (SNR). Also, the emission from the spark discharges without laser irradiance was recorded and used for background correction to mitigate the fraction of plasma emission that still could be detected.

### Electrics

The ignition system consists mainly of a coil. Direct current (DC) spark discharges are generated by this inductive configuration. The charging time of the primary coil (i.e., dwell time) was set to 3 ms, the upper limit, to maximize the generation of nickel atoms.

As illustrated in Figure 2b, two spark plugs (NGK Model BCP7ES) with nickel-based alloy electrodes were employed. For experimental purposes, both were manually modified by removing their original J-shaped side electrodes, resulting in the creation of two flat-top electrodes. These two spark plugs were individually mounted on a stage, which is designed for easy and independent exchanges of spark plugs for testing. The spark plug positioned in the upper location was connected to the ignition system, serving as the cathode. Conversely, the one located in the lower position was grounded and functioned as the anode. The stage was adjusted to position the two electrodes with a gap of 1.5 mm for an easier optical alignment.

## Results and Discussion

### Excitation Scan

The intensity of the LIF signal served as a primary indicator in our investigation. The wavelength of the excitation laser was first tuned in a wide range from 210 to 340 nm. From these preliminary results, a following excitation scan with higher spectral resolution was performed in a narrower range of 10 nm from 327.8 to 337.7 nm, with a 0.1 nm step size to determine the most optimal excitation wavelength.

**Figure 3.**
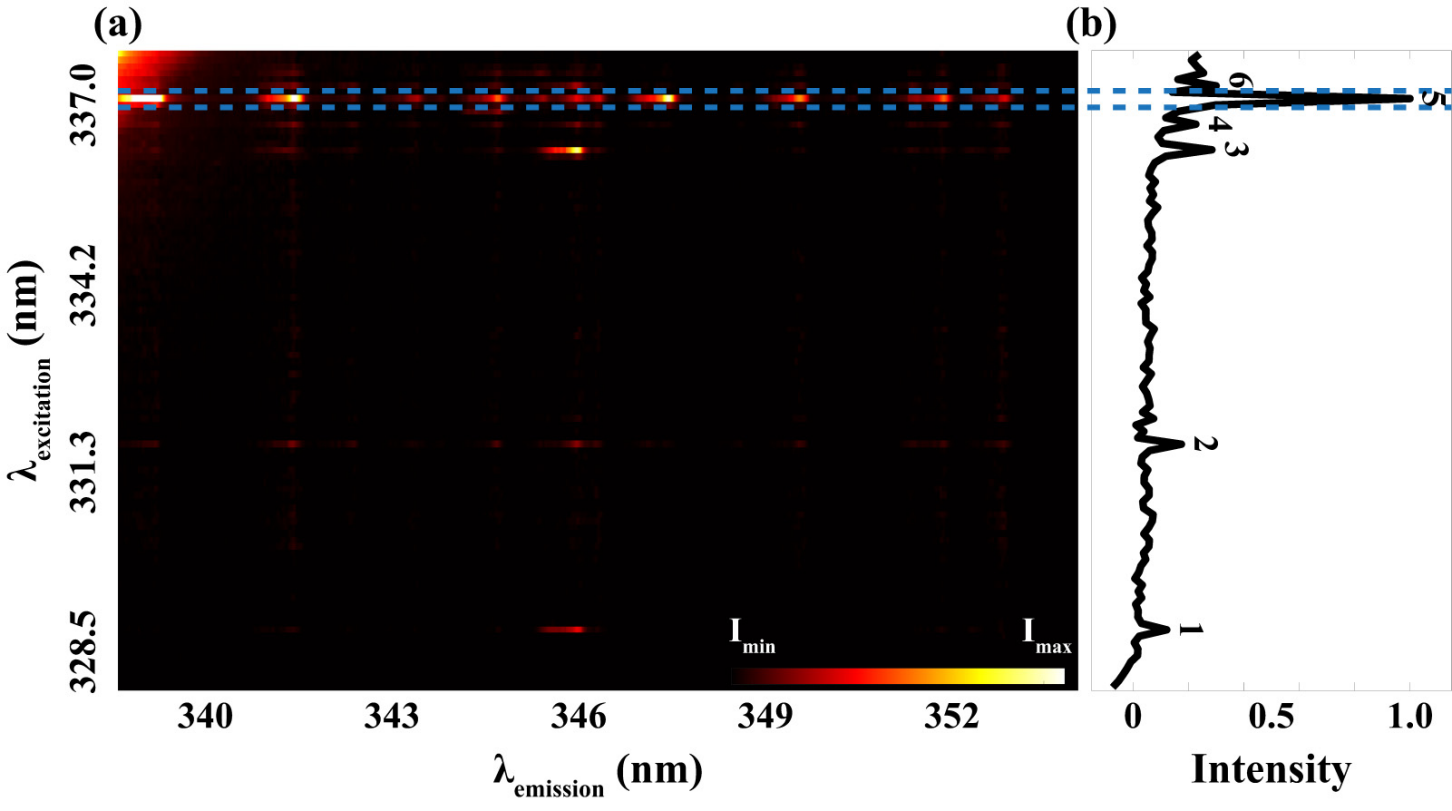
(a) Laser-induced fluorescence (LIF) signal from nickel atoms detected in the range 338.75–353.58 nm (*x*-axis) excited by the laser from 327.8 nm to 337.7 nm (*y*-axis). (b) Normalized excitation spectrum where peaks 1–6 are assigned for different excitation wavelengths.

Figure 3a is a synthesized plot based on all the recorded LIF signals with the excitation on the ordinate and the emission on the abscissa. Notably, the most robust fluorescence signals were observed when the excitation occurred at a wavelength of 337.0 nm. A normalized excitation spectrum is displayed in Figure 3b, obtained by a respective integration of emission signals detected at each excitation wavelength. In addition to the prominent peak at 337.0 nm, represented by peak 5, several other peaks were discerned. Also, the background exhibits an upward trend after peak 3, which can be attributed to the laser scattering inside the spectrometer.

In comparison with the Ni(I) data available in the NIST database (detailed in Table I), the six distinct peaks are identified at 328.7 nm, 331.6 nm, 336.2 nm, 336.6 nm, 337.0 nm, and 337.2 nm, respectively. It should be pointed out that peaks 4 and 5 seem to stem from two transitions that could not be resolved by our broadband picosecond laser system.

**Table I.** Peaks and corresponding transitions in Figure 3.^37^

Peak	λ_excitation_	*I*_measured_	λ_NIST_	*I*_NIST_ ^a^	Lower level			Upper level		
	(nm)		(nm)		Configuration	Term	*J*	Configuration	Term	*J*
1	328.7	0.122	328.695	–	3d^9^4s	^3^D	3	3d^9^4p	^3^F	2
2	331.6	0.175	331.566	0.228	3d^9^4s	^1^D	2	3d^9^4p	^1^F	3
3	336.2	0.285	336.156	0.114	3d^9^4s	^3^D	2	3d^9^4p	^3^F	2
4	336.6	0.227	336.577	0.114	3d^9^4s	^1^D	2	3d^8^4s4p	^3^F	3
			336.617	0.114	3d^8^4s^2^	^3^F	3	3d^9^4p	^1^F	
5	337.0	1.000	336.956	–	3d^8^4s^2^	^3^F	4	3d^9^4p	^3^D	3
			336.957	1.000^b^					*	
6	337.2	0.300	337.199	0.138	3d^8^4s^2^	^3^F	3	3d^8^4s4p	^3^G	4

^a^No data available in the NIST database, –.

^b^Highest relative intensity among the Ni(I) transitions in Table I in the NIST database.

As shown in Table I, the measured intensities (*I*_measured_) of the transitions are also compared with the intensities found in the NIST database (*I*_NIST_). The relative intensity notations in Table I are normalized against peak 5 which is the strongest from our measurements as well as the data from NIST. This transition is probed around 336.96 nm, and the spectral line is the strongest since pumping is carried out from the ground state of nickel which should be the most populated state among these conditions. Differences in peak intensities between our measurements and the data found in the NIST database can be observed, mainly resulting from the way the relative intensity was derived. The total fluorescence signal of a specific excitation wavelength (Figure 3b) is an accumulation of all emission peaks. It is important to note that the NIST database provides relative values calibrated to specific copper lines, derived from thermally excited optical emission using a 220 V, 10 A DC arc. These differing excitation sources can result in variations in relative peak intensities observed in the spectra.

### Energy Variation

**Figure 4.**
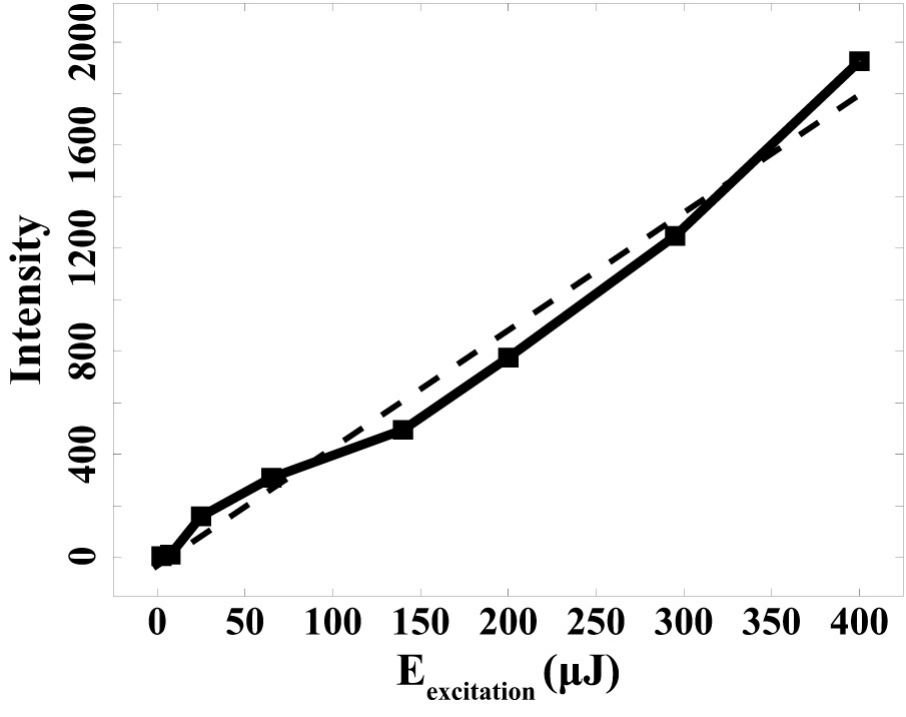
Fluorescence intensity dependency on excitation energy.

An examination of the fluorescence signal's dependence on excitation pulse energy was conducted next. The energy of excitation laser pulses at 337.0 nm was altered from 5 to 400 µJ. Figure 4 illustrates the outcome of this investigation. The slope observed in the energy dependence curves reveals that the fluorescence signal does not exhibit saturation behavior. Through the linear fitting, the coefficient of determination (R²) equals 0.983, affirming that under irradiation with an energy level of 400 µJ, the fluorescence signal still adheres to a linear regime.

### Spectroscopic Study

As revealed by the excitation scan results, the most intense fluorescence signal emerges when the excitation laser wavelength is centered at 337.0 nm. The spectral characteristic of corresponding LIF emissions is investigated here.

**Figure 5.**
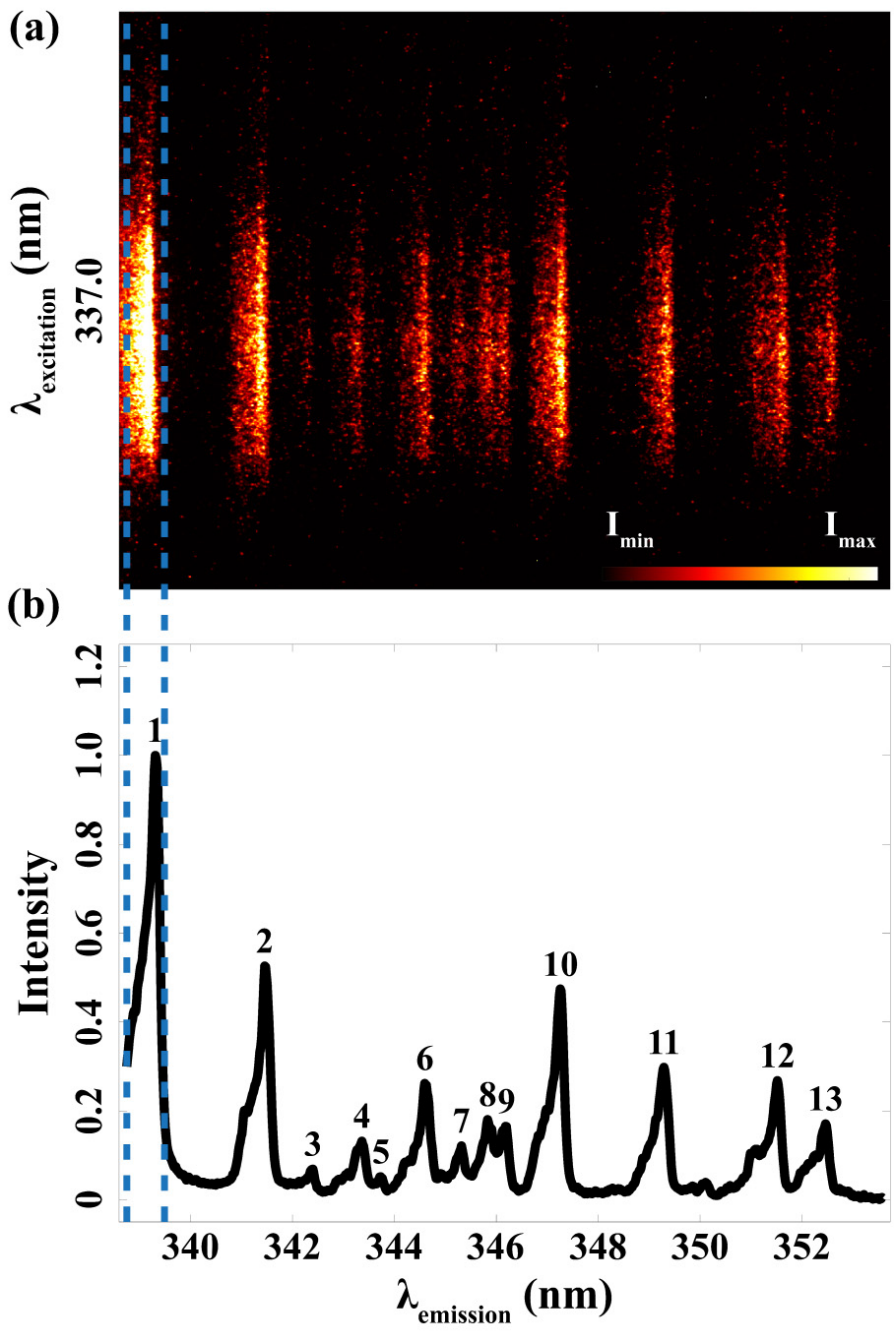
Laser-induced fluorescence signal excited by 337.0 nm and detected from 338.75 to 353.58 nm. (a) Acquired emission spectra. (b) Corresponding wavelengths after calibration.

Figure 5a is a representation of the fluorescence signal. The peaks in Figure 5b were identified as the spectral response from nickel using NIST data, which is displayed in detail in Table II. The measured emission spectral peaks with an average FWHM of 0.234 nm closely align with the wavelengths documented in the NIST database. This consistency clearly confirms that the observed fluorescence signal indeed originates from the vaporized nickel atoms stemming from the spark plug electrodes. Meanwhile, due to the resolution limit of the spectrometer, peaks 1, 2, 10, 12, and 13 are supposed to be a superposition of several transitions.

**Table II.** Peaks and corresponding transitions in Figure 5.^37^

Peak	λ_measured_	*I*_measured_	λ_NIST_	*I*_NIST_^a^	Lower level			Upper level		
	(nm)		(nm)		Configuration	Term	*J*	Configuration	Term	*J*
1	339.31	1.000	339.105	0.159	3d^8^4s^2^	^3^F	4	3d^9^4p	^3^F	4
			339.299	0.402	3d^9^4s	^3^D	3		^3^D	3
2	341.47	0.526	340.958	0.016	3d^8^4s^2^	^3^F	4	3d^9^4p	^3^F	3
			341.348	0.040			3			2
			341.394	0.040	3d^9^4s	^3^D	2	3d^8^4s4p	^5^F	2
			341.476	1.000^b^			3	3d^9^4p	^3^F	4
3	342.40	0.068	342.371	0.195	3d^9^4s	^3^D	1	3d^9^4p	^3^D	1
4	343.36	0.132	343.356	0.317	3d^9^4s	^3^D	3	3d^9^4p	^3^F	3
5	343.73	0.052	343.728	0.121	3d^8^4s^2^	^3^F	4	3d^8^4s4p	^5^F	4
6	344.60	0.262	344.626	0.585	3d^9^4s	^3^D	2	3d^9^4p	^3^D	2
7	345.32	0.121	345.289	0.159	3d^9^4s	^3^D	2	3d^8^4s4p	^5^F	3
8	345.83	0.180	345.847	0.610	3d^9^4s	^3^D	1	3d^9^4p	^3^F	2
9	346.18	0.165	346.165	0.610	3d^9^4s	^3^D	3	3d^8^4s4p	^5^F	4
10	347.26	0.474	346.750	0.024	3d^8^4s^2^	^3^F	3	3d^8^4s4p	^5^F	2
			346.949	0.029			2	3d^9^4p	^1^F	3
			347.254	0.195	3d^9^4s	^3^D	2		^3^D	3
11	349.29	0.298	349.296	0.671	3d^9^4s	^3^D	2	3d^9^4p	^3^P	1
12	351.52	0.268	351.034	0.317	3d^9^4s	^3^D	1	3d^9^4p	^3^P	0
			351.393	0.032			1	3d^8^4s4p	^5^F	2
			351.505	0.805			2	3d^9^4p	^3^F	3
13	352.46	0.171	351.977	0.080	3d^8^4s^2^	^3^F	2	3d^9^4p	^3^F	2
			352.307	–	3d^9^4s	^1^D	2	3d^8^4s4p	^3^G	3
			352.344	–		^3^D	3		^5^G	3
			352.454	1.000^a^			3	3d^9^4p	^3^P	2

^a^No data available in the NIST database, –.

^b^Highest relative intensity among all Ni(I) transitions in the NIST database.

**Figure 6.**
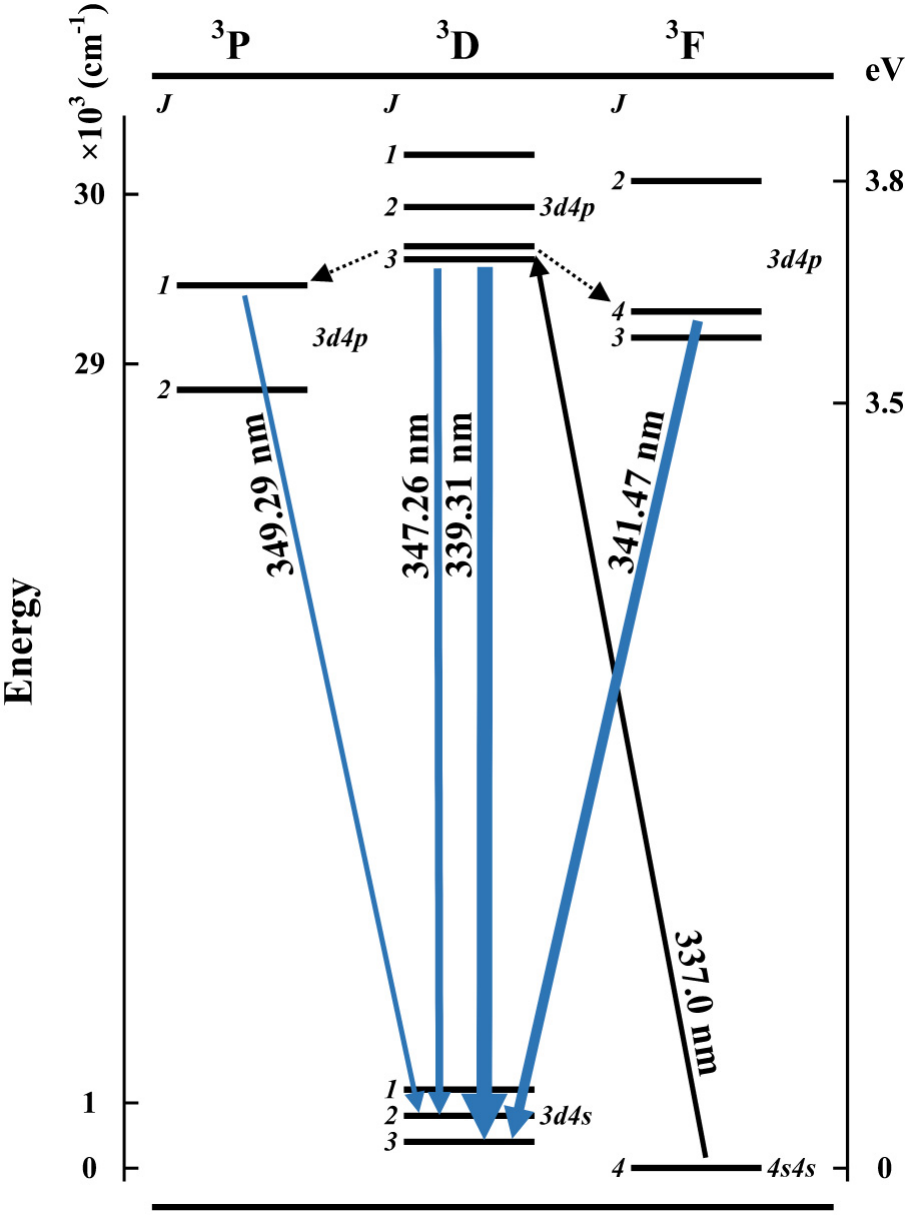
A simplified diagram of nickel energy levels with the four most intense fluorescence as well as the optimal excitation.

Similar to the abovementioned excitation scan, a comparison between the measured intensities (*I*_measured_) and the intensities in the NIST database (*I*_NIST_) is also shown in Table II. The highest relative intensity is set as 1.000 for normalization to show the different distribution of transitions due to the two excitation sources. The strongest transition observed in our experiment occurs at 339.31 nm, which is 37.3% of the total intensity of all emissions. Illustrated in Figure 6, this transition shares the same upper state as the excitation at 337.0 nm, which makes a high probability of de-excitation. Sharing the same reason, the intensity of peak 10 (347.26 nm) is much stronger than the values in NIST. About the other two transitions, transferring to the two closest energy levels due to collision, 341.47 and 349.29 nm have the second and fourth strongest fluorescence intensity.

The investigation shows that the LIF technique works well for the detection of evaporated nickel atoms in the presence of intense background radiation, and it has great potential for in situ studies of electrode wear of spark plugs in more realistic operation conditions, such as in a high-pressure chamber.

Spectral analysis shows that the LIF signals of nickel are different from the strong emissions of common radicals that could be generated in plasma, such as hydroxide (OH) (310 nm)^13^ and nitric oxide (NO) (230–260 nm).^38^ Accordingly, employing a narrower detection window, for instance by integrating a bandpass filter, can further enhance the SNR by excluding interference from the excitation laser and eliminating unwanted signal contributions from other species or N_2_ plasma. Deserves to be mentioned that the predominant emission detected at 339.31 nm is proximate to the optimal excitation wavelength of 337.0 nm, posing a risk of being partly cut off due to the unsharp transmission curve of bandpass filters. From our spectroscopic study, it is indicated that over 60% of the total intensity will still be retained.

In addition, an initial study of the excitation energy variation has shown that the fluorescence signal is in a linear regime under 400 µJ. From this finding, it is evident that the fluorescence quantum yield is quite low due to collisional quenching. It may thus be possible to increase the laser pulse energy and perform a more narrowband excitation to increase SNRs without reaching saturation. A more advantageous option would be to use a neodymium-doped yttrium aluminum garnet (Nd:YAG) pumped dye laser system, which has a higher energy and a narrower bandwidth (∼0.1 cm^−1^ FWHM) compared to the picosecond laser system (∼10 cm^−1^ FWHM). The increased pulse energy and energy spectral density would further enhance the sensitivity and selectivity of nickel detection.

Although our work shows that both excitation and emissions of nickel atoms do not overlap with some common species that appeared in combustion, such as methylidyne (CH), OH, hydrogen carbonate (HCO), and formaldehyde (CH_2_O),^12^ many other species could exist and may also emit interfering luminescence or even absorb the excitation energy. Thus, more careful consideration needs to be taken for the utilization of the LIF technique in a real engine operation.

## Conclusion

This paper presents an investigation of nickel atoms that evaporate from spark plug electrodes during the discharges using LIF. Excitation radiation was provided by a tunable picosecond laser system. After calibration of the wavelengths of the detected fluorescence signal and comparison against the NIST database, it could be verified that the emission spectra originate from the nickel atoms eroded from the electrodes. The most favorable transition for excitation of nickel is determined at ∼336.96 nm and the corresponding emission with the highest intensity is detected at 339.31 nm. The optical investigations of nickel atoms in terms of both excitation and emission do not overlap with some common radicals in plasma discharges, such as OH and NO. Consequently, what we performed under ambient conditions can most likely be adapted for use in more realistic settings, such as a high-pressure chamber. An investigation of the relationship between the fluorescence intensity and the excitation energy was carried out. It was found that the LIF signal is still located in the linear regime. The signal can be further increased by higher excitation energy and hence enhance the sensitivity and selectivity of nickel detection. The authors believe that this strategy for the detection of nickel atoms using LIF can also be applied to other metal atoms that are commonly seen in the alloys of spark plug electrodes, such as copper (Cu), platinum (Pt), and iridium (Ir).

## References

[1] European Union (EU). “Regulation (EU) 2018/1999 of the European Parliament and of the Council of 11 December 2018 on the Governance of the Energy Union and Climate Action”. https://eur-lex.europa.eu/eli/reg/2018/1999/oj [accessed Sep 4 2024].

[2] European Union (EU). “Regulation (EU) 2021/1119 of the European Parliament and of the Council of 30 June 2021 Establishing the Framework for Achieving Climate Neutrality and Amending Regulations (EC) No 401/2009 and (EU) 2018/1999 (‘European Climate Law’)”. https://eur-lex.europa.eu/legal-content/EN/TXT/?toc=OJ%3AL%3A2021%3A243%3ATOC&uri=uriserv%3AOJ.L_.2021.243.01.0001.01.ENG [accessed Sep 4 2024].

[3] Mahendar S.K. Erlandsson A. Adlercreutz L. . “Challenges for Spark Ignition Engines in Heavy Duty Application: A Review”. SAE International. 2018.

[4] Awad O.I. Mamat R. Ali O.M. Sidik N.A.C. , et al. “Alcohol and Ether as Alternative Fuels in Spark Ignition Engine: A Review”. Renew. Sustain. Energy Rev. 2018. 82(Part 3): 2586–2605.

[5] Javan S. Hosseini S.V. Alaviyoun S.S. Ommi F. . “Effect of Electrode Erosion on the Required Ignition Voltage of Spark Plug in CNG Spark Ignition Engine”. J. Engine Res. 2012. 26(26): 31–39.

[6] Lin H.T. Brady M.P. Richards R.K. Layton D.M. . “Characterization of Erosion and Failure Processes of Spark Plugs After Field Service in Natural Gas Engines”. Wear. 2005. 259(7–12): 1063–1067.

[7] Różowicz S. . “Use of the Mathematical Model of the Ignition System to Analyze the Spark Discharge, Including the Destruction of Spark Plug Electrodes”. Open Phys. 2018. 16(1): 57–62.

[8] Javan S. Alaviyoun S.S. Hosseini S.V. Ommi F. . “Experimental Study of Fine Center Electrode Spark Plug in Bi-Fuel Engines”. J. Mech. Sci. Technol. 2014. 28(3): 1089–1097.

[9] Klimstra J. Overmars F. . “Monitoring the Spark-Plug Gap of Natural-Gas-Fuelled Stationary Engines”. SAE Trans.: J. Fuels Lubricants. 1991. 100(Section 4): 945–956. http://www.jstor.org/stable/44553647 [accessed Sep 4 2024].

[10] Kimbara Y. Noguchi Y. Ishiguro T. . “Study on Spark Plug Carbon Fouling”. SAE Transactions”. SAE Trans. 1980. 89(Section 3): 2492–2502. https://www.jstor.org/stable/44729867 [accessed Sep 4 2024].

[11] Gray E.W. Pharney J.R. . “Electrode Erosion by Particle Ejection in Low-Current Arcs”. J. Appl. Phys. 1974. 45(2): 667–671.

[12] Aldén M. . “Spatially and Temporally Resolved Laser/Optical Diagnostics of Combustion Processes: From Fundamentals to Practical Applications”. Proc. Combust. Inst. 2023. 39(1): 1185–1228.

[13] Bao Y. Dorozynska K. Stamatoglou P. Kong C. , et al. “Single-Shot 3D Imaging of Hydroxyl Radicals in the Vicinity of a Gliding Arc Discharge”. Plasma Sources Sci. Technol. 2021. 30(4): 04LT04.

[14] Barnat E.V. . “Multi-Dimensional Optical and Laser-Based Diagnostics of Low-Temperature Ionized Plasma Discharges”. Plasma Sources Sci. Technol. 2011. 20(5): 053001.

[15] Aldén M. Edner H. Svanberg S. . “Coherent Anti-Stokes Raman Spectroscopy (Cars) Applied in Combustion Probing”. Phys. Scr. 1983. 27(1): 29–38.

[16] Hobbs S.E. Olesik J.W. . “Laser-Excited Fluorescence Studies of Matrix-Induced Errors in Inductively Coupled Plasma Spectrometry: Implications for ICP–Mass Spectrometry”. Appl. Spectrosc. 1991. 45(9): 1395–1407.

[17] Simeonsson J.B. Ng K.C. Winefordner J.D. . “Single- and Double-Resonance Atomic Fluorescence Spectrometry with Inductively Coupled Plasma Atomization and Laser Excitation”. Appl. Spectrosc. 1991. 45(9): 1456–1462.

[18] Simeonsson J.B. Ng K.C. Winefordner J.D. . “Laser-Induced Fluorescence of Rare Earth Elements in the Inductively Coupled Plasma”. Anal. Chim. Acta. 1992. 258(1): 73–81.

[19] Baiocchi C. Giacosa D. Saini G. Cavalli P. , et al. “Determination of Thallium in Antarctic Snow by Means of Laser Induced Atomic Fluorescence and High Resolution Inductively Coupled Plasma Mass Spectrometry”. Int. J. Environ. Anal. Chem. 1994. 55(1–4): 211–218.

[20] Olesik J.W. . “Fundamental Research in ICP-OES and ICPMS”. Anal. Chem. 1996. 68(15): 469–474.

[21] Olesik J.W. Kinzer J.A. McGowan G.J. . “Observation of Atom and Ion Clouds Produced from Single Droplets of Sample in Inductively Coupled Plasmas by Optical Emission and Laser-Induced Fluorescence Imaging”. Appl. Spectrosc. 1997. 51(5): 607–616.

[22] Simeonsson J.B. Ezer M. Pacquette H.L. Preston S.L. Swart D.J. . “Laser-Induced Fluorescence of As, Se, and Sb in the Inductively Coupled Plasma”. Spectrochim. Acta, Part B. 1997. 52(13): 1955–1963.

[23] Lehn S.A. Huang M. Warner K.A. Gamez G. Hieftje G.M. . “Spatially Resolved Ground-State Number Densities of Calcium and Strontium Ion in an Inductively Coupled Plasma in Contact with an Inductively Coupled Plasma Mass Spectrometry Sampling Interface”. Spectrochim. Acta. Part B. 2003. 58(9): 1647–1662.

[24] Mills A.A. Macedone J.H. Farnsworth P.B. . “High Resolution Imaging of Barium Ions and Atoms Near the Sampling Cone of an Inductively Coupled Plasma Mass Spectrometer”. Spectrochim. Acta, Part B. 2006. 61(9): 1039–1049.

[25] Nishioka S. Hanashi K. Okabe S. . “Super Ignition Spark Plug with Wear Resistive Electrode”. SAE Technical Paper. 2008. Pp. 2008-01–0092.

[26] Lins G. Hartmann W. . “Metal Vapor Densities and Excitation Temperatures in a Pseudospark Switch”. IEEE Trans. Plasma Sci. 1993. 21(5): 511–515.

[27] Marunkov A. Chekalin N. Enger J. Axner O. . “Detection of Trace Amounts of Ni by Laser-Induced Fluorescence in Graphite Furnace with Intensified Charge Coupled Device”. Spectrochim. Acta, Part B. 1994. 49(12–14): 1385–1410.

[28] Epstein M.S. Bradshaw J. Bayer S. Bower J. , et al. “Application of Laser-Excited Atomic Fluorescence Spectrometry to the Determination of Nickel and Tin”. Appl. Spectrosc. 1980. 34(3): 372–376.

[29] Li J. Hao Z. Zhao N. Zhou R. , et al. “Spatially Selective Excitation in Laser-Induced Breakdown Spectroscopy Combined with Laser-Induced Fluorescence”. Opt. Express. 2017. 25(5): 4945. 28380761 10.1364/OE.25.004945

[30] Li J. . “Determination of Molybdenum, Nickel, and Chromium in Low-Alloy Steels Using Laser-Induced Breakdown Spectroscopy Assisted with Laser-Induced Fluorescence and Discrete Wavelet Transform”. At. Spectrosc. 2023. 44(04): 219–226.

[31] De Boer G. Arepalli S. Holmes W. Nikolaev P. , et al. “Detection of Nickel Atom by Laser Induced Fluorescence During Carbon Nanotube Formation in a Laser Produced Plume”. J. Appl. Phys. 2001. 89(10): 5760–5768.

[32] Dorval N. Foutel-Richard A. Cau M. Loiseau A. , et al. “In-Situ Optical Analysis of the Gas Phase During the Formation of Carbon Nanotubes”. J. Nanosci. Nanotechnol. 2004. 4(4): 450–462. 15296236 10.1166/jnn.2004.060

[33] Cau M. Dorval N. Cao B. Attal-Trétout B. , et al. “Spatial Evolutions of Co and Ni Atoms During Single-Walled Carbon Nanotubes Formation: Measurements and Modeling”. J. Nanosci. Nanotechnol. 2006. 6(5): 1298–1308. 16792356 10.1166/jnn.2006.178

[34] Cau M. Dorval N. Attal-Trétout B. Cochon J.-L. , et al. “Formation of Carbon Nanotubes: In Situ Optical Analysis Using Laser-Induced Incandescence and Laser-Induced Fluorescence”. Phys. Rev. B. 2010. 81(16): 165416 (1–22).

[35] Kim W. Bae C Michler T Toedter O Koch T . “Spatio-Temporally Resolved Emission Spectroscopy of Inductive Spark Ignition in Atmospheric Air Condition”. In: Gunther M. Sens M , editors. Ignition Systems for Gasoline Engines. Third International Conference Proceedings. Berlin, Germany, 3–4 November 2016. Pp. 209–221.

[36] Ni C. Cheng X. . “Ab Initio Study of the Second Positive System of N_2_ at High Temperature”. Comput. Theor. Chem. 2021. 1197: 113158.

[37] Kramida A. Ralchenko Yu. Reader J. , et al. “NIST Atomic Spectra Database (Version 5.11)”. https://www.nist.gov/pml/atomic-spectra-database [accessed Sep 4 2024].

[38] Machala Z. Janda M. Hensel K. Jedlovský I. , et al. “Emission Spectroscopy of Atmospheric Pressure Plasmas for Bio-Medical and Environmental Applications”. J. Mol. Spectrosc. 2007. 243(2): 194–201.

